# Impact of oral rehabilitation on the quality of life of partially dentate elders in a randomised controlled clinical trial: 2 year follow-up

**DOI:** 10.1371/journal.pone.0203349

**Published:** 2018-10-11

**Authors:** Gerald McKenna, Patrick Finbarr Allen, Martina Hayes, Cristiane DaMata, Ciaran Moore, Michael Cronin

**Affiliations:** 1 Centre for Public Health, Queen’s University Belfast, Belfast, Northern Ireland; 2 Faculty of Dentistry, National University of Singapore, Singapore, Singapore; 3 Cork University Dental School and Hospital, University College Cork, Cork, Ireland; 4 School of Mathematics, University College Cork, Ireland; Janssen Research and Development, UNITED STATES

## Abstract

**Objective:**

This randomised clinical trial aimed to compare the impact of two different tooth replacement strategies for partially dentate older patients namely; removable partial dentures (RPDs) and functionally orientated treatment based on the shortened dental arch (SDA) concept, on Oral Health-related Quality of Life (OHrQOL).

**Methods:**

89 patients completed a randomised clinical trial. Patients were recruited in two centres: Cork University Dental Hospital (CUDH) and a Geriatric Day Hospital (SFDH). 44 patients were randomly allocated to the RPD group and 45 to the SDA group where adhesive bridgework was used to provide 10 pairs of occluding contacts. The impact of treatment on OHrQOL was used as the primary outcome measure. Each patient completed the Oral Health Impact Profile (OHIP-14) at baseline, 1, 6, 12 and 24 months after treatment.

**Results:**

Both treatment groups reported improvements in OHIP-14 scores at 24 months (p<0.05). For the SDA group OHIP-14 scores improved by 8.0 scale points at 12 months (p<0.001) and 5.9 scale points at 24 months (p<0.05). For the RPD group OHIP-14 scores improved by 5.7 scale points at 12 months (p<0.05) and 4.2 scale points at 24 months (p<0.05). Analysis using ANCOVA showed that there were significant between group differences recorded in both treatment centres. 24 months after intervention the SDA group recorded better OHIP-14 scores by an average of 2.9 points in CUDH (p<0.0001) and by an average of 7.9 points in SFDH (p<0.0001) compared to the RPD group.

**Conclusions:**

Patients in the SDA group maintained their improvements in OHrQOL scores throughout the 24 month study period. For the RPD group the initial improvement in OHrQOL score began to diminish after 6 months, particularly for those treated in SFDH. Thus, the benefits of functionally orientated treatment increased over time, particularly for the older, more systemically unwell cohort in SFDH.

## Introduction

Significant changes in the oral health of older patients have resulted in a partially dentate older population with decreased prevalence of edentulism [1]. In many counties, the current conventional care delivered to replace missing teeth for older patients involves provision of removable partial dentures (RPDs). However, less complex, functionally orientated treatment solutions are very applicable to partially dentate older patients including the Shortened Dental Arch (SDA) concept [2]. First described in 1981, the SDA approach aims to provide patients with a functional dentition of 10 occluding pairs of teeth or contacts without the need for a RPD [3]. Treatment efforts are focused on the anterior teeth which provides patients with an aesthetic dentition which can also be maintained successfully. Studies have shown that by providing 10 occluding pairs of teeth or contacts, patients can achieve suboptimal but acceptable levels of dental function [4,5]. Evidence has shown that use of the SDA concept in older patients can have positive impacts on nutritional status and is more cost-effective to deliver and maintain than RPDs [6,7]. However, evidence at the highest level remains extremely limited, with very few reported randomised trials reported.

Whilst a small number of patients will retain the 20 natural teeth necessary to achieve a SDA, it is more common for patients to be restored to a SDA. This can be done using a variety of fixed prosthodontic options including conventional bridgework, dental implants and adhesive resin bonded bridgework (RBB). RBB has been shown to be an effective and minimally invasive way of replacing missing teeth to provide patients with a SDA [8]. Despite the evidence in favour of the SDA concept it remains an underutilised approach [9].

The aim of this randomised clinical trial was to compare two different tooth replacement strategies for partially dentate older patients; namely functionally orientated treatment according to the principles of the shortened dental arch (SDA) and conventional treatment using removable partial dentures (RPDs). The primary outcome measure for this study was impact of the treatments on Oral Health-related Quality of Life (OHRQoL) measured using the short form of the Oral Health Impact Profile (OHIP-14). A secondary aim for this study was to report on the seven conceptional domains which make up summary OHIP-14 scores.

The null hypothesis for the study stated that patients treated according to the principles of the SDA would be no worse off than those treated using RPDs in terms of impact on OHRQoL.

## Methodology

The trial methodology has been described previously in other publications reporting on shorter follow up periods and secondary outcomes measures [2,6,7].

**Fig 1:**
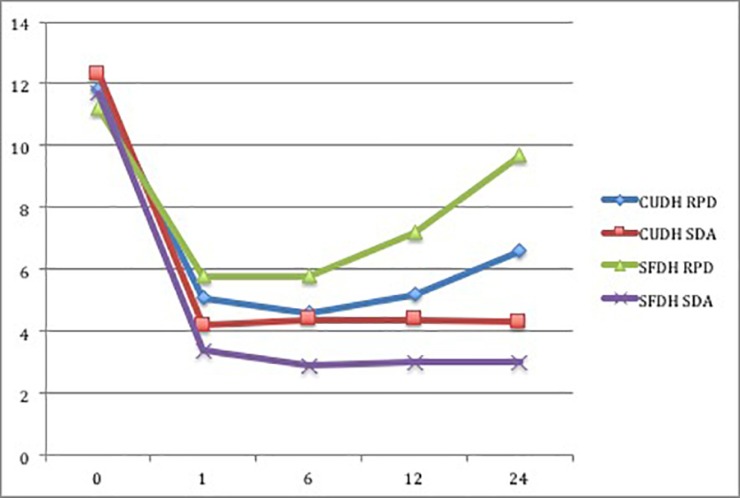
Patient flow diagram.

As illustrated in Fig 1, a randomised controlled clinical trial (RCT) was conducted. Patients were recruited from two centres: Cork University Dental Hospital (CUDH) and St Finbarr's Geriatric Day Hospital (SFDH) in Cork, Ireland. Recruitment ran from January 2011 until March 2013. Two year follow up data was collected from the last patient in May 2015. Patients were included in the study if they were 65 years or older and seeking replacement of missing natural teeth. Participants had a minimum of six remaining natural teeth in both arches of good prognosis, could accept routine dental care in a dental chair, could communicate in English and had no medical conditions which precluded routine dental treatment. Full ethical approval was granted for the study from the Cork Teaching Hospitals Ethics Committee (ref: ECM 5 (9) 05/02/08 S1 Fig). Each patient was provided with written information detailing the proposed treatment involved and each patient completed a written consent form prior to treatment. A power calculation was made based on summary OHIP-14 score data from the United Kingdom Adult Dental Health Survey [10]. The calculation was based on demonstrating that mean OHIP-14 in the RPD group was no worse than in the SDA group by a maximum of 4.0. The standard deviation used was 7.4, power was set at 80% with a one sided 5% level of significance. The power calculation indicated that 44 patients were required to complete the study from both treatment groups. The attrition rate was set at 30% to allow for drop outs during the study, so the targeted baseline recruitment was 130.

Randomisation was performed using a computer generated schedule in SAS^®^. Patients were randomly allocated to two different treatment groups: the RPD group and the SDA group. Randomisation was conducted in blocks of varying length and was stratified according to age and gender. Separate randomisation schedules were generated for both recruitment site and the treatment groups included patients recruited from both centres, randomised independently. Patient randomisation was conducted by a research assistant and the allocation was concealed from the clinical operator. Initially, all patients received standardised dental care to render them dentally fit including extraction of hopeless teeth, restoration of caries and non surgical management of periodontal disease.

Patients from each treatment group received standardised care according to a treatment protocol. Each patient from the RPD group had all missing natural teeth replaced with RPDs fabricated with cobalt–chromium frameworks. Each patient from the SDA group was restored to a premolar occlusion of 10 occluding pairs of natural and replacement teeth using RBB throughout the arch. Posterior teeth distal to the SDA were left unopposed. The RBB was provided using a standardised protocol in each case. Each item of fixed and removable prosthodontics was constructed by the same dental laboratory. All operative treatment was conducted by a single operator with postgraduate training in clinical prosthodontics.

OHRQoL was measured using the OHIP-14 questionnaire [11]. The measure contains statements divided into seven theoretical domains, namely functional limitation, pain, psychological discomfort, physical disability, psychological disability, social disability and, handicap. As a cumulative record of negative impacts a reduction in OHIP-14 score represents an improvement in OHRQoL. The questionnaire was administered by a research nurse at baseline, 1 month, 6 months, 12 months and 24 months after treatment intervention. The research nurse was blinded to the treatment group allocation of all patients. Systemic comorbidity was recorded for each patient by a trained medical professional using the modified Cumulative Illness Rating Scale (CIRS) at baseline [12]. A measurement of social class was made for each patient using their longest held occupation. Patients were categorised according to the Registrar General's Social Classification [13].

### Statistical analysis

All variables recorded were summarised using appropriate descriptive statistics and graphics. Relationships between the treatment groups and mean summary OHIP-14 and OHIP-14 domain scores were assessed using linear models and logistic regression (binary and ordinal) models. Patient demographic variables (including age, gender, recruitment site, modified CIRS score and Social Class) were controlled for by including them in these models as covariates/factors. All variables recorded were presented by time-point and by treatment group. All patients were analysed according to initial treatment intent (determined by the randomisation process), not the treatment eventually administered. This was designed to reduce bias and to maintain the integrity of the randomisation process (intention-to-treat analysis).

The trial was registered retrospectively on ISRCTN (Registration number: 26302774). The authors confirm that all ongoing and related trials for this intervention are also registered.

## Results

The baseline characteristics of the study participants are presented in Table 1.

**Table 1:** Baseline characteristics of study participants.

	Patient Demographics																	
	Gender				Age		Social Class										Comorbidity	
	Male		Female				I		II		III		IV		V		CIRS Score	
	n	%	n	%	Mean (Years)	SD	n	%	n	%	n	%	n	%	n	%	Median	IQR
Treatment Group																		
RPD	29	44.6	36	55.4	74.1	6.2	2	3.1	18	27.7	25	38.5	10	15.4	9	13.9	15	12
SDA	29	43.3	38	56.7	73.9	7.0	6	16.4	11	16.4	24	35.8	11	16.4	13	19.4	17	17

### OHIP-14 summary scores

**Fig 2:**
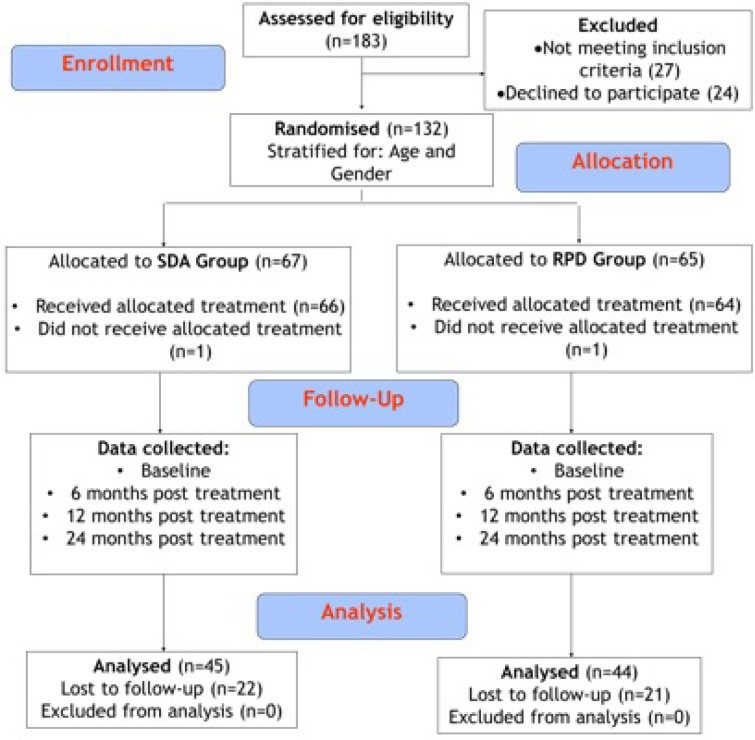
Mean OHIP-14 summary scores for all participants.

After 24 months, 89 participants completed the randomised controlled clinical trial. The mean OHIP-14 summary score for all patients are illustrated in Table 2 and Fig 2. A mixed model analysis of covariance (ANCOVA) for repeated measures was fitted to OHIP-14 summary scores. Fixed factors in the model were treatment group, time point (1, 6, 12 or 24 months), treatment centre, social class and gender. Within the model the covariates used were the baseline values, comorbidity scores and age (Table 3). The two-level interactions between treatment group and each of time point, treatment centre, social class, gender, comorbidity scores and age were considered for inclusion. These were retained in the model only if significant at the 5% level of significance. Relevant three-level interactions based on the significant two-level interactions were also considered for inclusion. These were to be retained in the model only if significant at the 5% level of significance. The model assumptions were checked using residual analyses. Statistical analysis was performed using SAS® version 9.3 (SAS Institute Inc., Cary, North Carolina, USA).

**Table 2:** OHIP-14 scores recorded for all study participants.

			Time Point														
			0			1			6			12			24		
			n	Mean	SD	n	Mean	SD	n	Mean	SD	n	Mean	SD	n	Mean	SD
**Summary OHIP-14 Score**	**Centre**	** **	40	11.8	4.5	35	5.1	3.5	32	4.6	3.2	32	5.2	3.5	32	6.6	3.2
	**CUDH**	**RPD**															
		**SDA**	40	12.3	5.9	34	4.2	3.2	33	4.4	3.2	33	4.4	2.9	33	4.3	2.6
		**Total**	80	12.0	5.2	69	4.7	3.4	65	4.5	3.1	65	4.8	3.2	65	5.4	3.1
	**SFDH**	**RPD**	25	11.2	5.0	18	5.8	2.7	13	5.8	3.1	13	7.2	3.1	12	9.7	2.7
		**SDA**	27	11.7	4.9	18	3.4	2.4	14	2.9	2.0	14	3.0	1.4	12	3.0	1.1
		**Total**	52	11.4	4.9	36	4.6	2.8	27	4.3	3.0	27	5.0	3.2	24	6.3	4.0
	**Total**	**RPD**	65	11.5	4.7	53	5.4	3.3	45	4.9	3.2	45	5.8	3.5	44	7.4	3.3
		**SDA**	67	12.0	5.5	52	4.0	2.9	47	3.9	2.9	47	4.0	2.6	45	4.0	2.3
		**Total**	132	11.8	5.1	105	4.7	3.2	92	4.4	3.1	92	4.8	3.2	89	5.7	3.3
**Functional Limitation**	**Centre**	** **	40	1.2	1.4	35	0.5	0.7	32	0.4	0.7	32	0.6	0.8	32	1.0	1.0
	**CUDH**	**RPD**															
		**SDA**	40	1.4	1.6	34	0.5	1.0	33	0.5	1.0	33	0.5	1.0	33	0.6	0.9
		**Total**	80	1.3	1.5	69	0.5	0.8	65	0.5	0.8	65	0.5	0.9	65	0.8	0.9
	**SFDH**	**RPD**	25	1.4	1.5	18	0.7	1.0	13	0.4	0.7	13	0.5	0.9	12	0.8	0.9
		**SDA**	27	1.1	1.2	18	0.3	0.6	14	0.4	0.6	14	0.4	0.6	12	0.6	0.5
		**Total**	52	1.3	1.3	36	0.5	0.8	27	0.4	0.6	27	0.4	0.8	24	0.7	0.8
	**Total**	**RPD**	65	1.3	1.4	53	0.6	0.8	45	0.4	0.7	45	0.5	0.8	44	0.9	0.9
		**SDA**	67	1.3	1.4	52	0.4	0.8	47	0.5	0.9	47	0.5	0.9	45	0.6	0.8
		**Total**	132	1.3	1.4	105	0.5	0.8	92	0.4	0.8	92	0.5	0.8	89	0.8	0.9
**Physical Pain**	**Centre**	** **	40	3.1	1.7	35	2.0	1.4	32	1.9	1.5	32	2.0	1.4	32	2.3	1.3
	**CUDH**	**RPD**															
		**SDA**	40	2.7	1.4	34	1.5	1.5	33	1.5	1.5	33	1.6	1.4	33	1.6	1.2
		**Total**	80	2.9	1.6	69	1.7	1.5	65	1.7	1.5	65	1.8	1.4	65	2.0	1.3
	**SFDH**	**RPD**	25	3.2	1.9	18	2.6	1.5	13	3.2	1.4	13	3.5	1.2	12	3.7	1.2
		**SDA**	27	3.2	1.6	18	1.8	1.2	14	1.4	1.2	14	1.6	1.0	12	1.8	1.0
		**Total**	52	3.2	1.7	36	2.2	1.4	27	2.2	1.6	27	2.5	1.4	24	2.7	1.4
	**Total**	**RPD**	65	3.2	1.8	53	2.2	1.5	45	2.3	1.6	45	2.4	1.5	44	2.7	1.4
		**SDA**	67	2.9	1.5	52	1.6	1.4	47	1.5	1.4	47	1.6	1.3	45	1.6	1.2
		**Total**	132	3.0	1.6	105	1.9	1.5	92	1.9	1.5	92	2.0	1.4	89	2.2	1.4
**Psychological Discomfort**	**Centre**	** **	40	2.8	1.7	35	1.1	1.1	32	1.0	1.1	32	1.2	1.3	32	1.2	1.3
	**CUDH**	**RPD**															
		**SDA**	40	3.3	2.1	34	0.9	1.1	33	0.9	1.1	33	0.9	1.0	33	0.8	0.9
		**Total**	80	3.0	1.9	69	1.0	1.1	65	0.9	1.1	65	1.0	1.1	65	1.0	1.1
	**SFDH**	**RPD**	25	2.3	1.9	18	0.8	1.1	13	0.5	1.0	13	1.5	1.7	12	1.3	1.6
		**SDA**	27	2.6	1.7	18	0.5	0.9	14	0.4	0.7	14	0.3	0.7	12	0.2	0.6
		**Total**	52	2.4	1.8	36	0.6	1.0	27	0.4	0.8	27	0.9	1.4	24	0.8	1.3
	**Total**	**RPD**	65	2.6	1.8	53	1.0	1.1	45	0.8	1.1	45	1.3	1.4	44	1.2	1.3
		**SDA**	67	3.0	2.0	52	0.7	1.0	47	0.7	1.0	47	0.7	0.9	45	0.6	0.9
		**Total**	132	2.8	1.9	105	0.9	1.1	92	0.8	1.1	92	1.0	1.2	89	0.9	1.2
**Physical Disability**	**Centre**	** **	40	1.9	1.3	35	0.7	0.9	32	0.7	0.9	32	0.8	1.0	32	1.0	1.0
	**CUDH**	**RPD**															
		**SDA**	40	2.0	1.7	34	0.6	1.0	33	0.6	1.0	33	0.5	0.8	33	0.5	0.8
		**Total**	80	2.0	1.5	69	0.7	0.9	65	0.6	0.9	65	0.6	0.9	65	0.8	0.9
	**SFDH**	**RPD**	25	1.7	1.5	18	1.3	1.1	13	1.3	1.3	13	1.4	1.4	12	2.0	1.0
		**SDA**	27	2.4	1.2	18	0.6	0.8	14	0.6	0.7	14	0.6	0.6	12	0.4	0.5
		**Total**	52	2.1	1.4	36	1.0	1.0	27	1.0	1.1	27	1.0	1.1	24	1.2	1.1
	**Total**	**RPD**	65	1.8	1.4	53	0.9	1.0	45	0.9	1.0	45	1.0	1.1	44	1.3	1.1
		**SDA**	67	2.1	1.5	52	0.6	0.9	47	0.6	0.9	47	0.5	0.8	45	0.5	0.8
		**Total**	132	2.0	1.5	105	0.8	1.0	92	0.7	1.0	92	0.7	1.0	89	0.9	1.0
**Psychological Disability**	**Centre**	** **	40	2.5	1.9	35	0.8	1.1	32	0.6	0.9	32	0.6	1.0	32	1.1	0.9
	**CUDH**	**RPD**															
		**SDA**	40	2.5	1.8	34	0.8	1.1	33	0.8	1.1	33	0.8	1.1	33	0.8	1.0
		**Total**	80	2.5	1.8	69	0.8	1.1	65	0.7	1.0	65	0.7	1.0	65	1.0	1.0
	**SFDH**	**RPD**	25	2.1	1.9	18	0.4	0.7	13	0.5	0.8	13	0.5	0.8	12	1.8	0.6
		**SDA**	27	2.1	1.8	18	0.2	0.7	14	0.1	0.3	14	0.1	0.3	12	0.1	0.3
		**Total**	52	2.1	1.8	36	0.3	0.7	27	0.3	0.6	27	0.3	0.6	24	1.0	1.0
	**Total**	**RPD**	65	2.4	1.9	53	0.6	1.0	45	0.6	0.9	45	0.6	0.9	44	1.3	0.9
		**SDA**	67	2.3	1.8	52	0.6	1.0	47	0.6	1.0	47	0.6	1.0	45	0.6	0.9
		**Total**	132	2.4	1.8	105	0.6	1.0	92	0.6	0.9	92	0.6	0.9	89	1.0	1.0

**Table 3:** Mixed model analysis of covariance (ANCOVA) for repeated measures fitted to OHIP-14 summary scores.

Effect	Numerator DF	Denominator DF	F Value	P-value
**Treatment Group**	1	261	32.7	< 0.0001
**Baseline Score**	1	261	21.9	< 0.0001
**Time Point**	3	261	24.8	< 0.0001
**Treatment Centre**	1	261	0.4	0.5271
**Social Class**	5	261	0.5	0.7923
**Gender**	1	261	0.7	0.4217
**Comorbidity Score**	1	261	1.5	0.2287
**Age**	1	261	0.7	0.4199
Group*Time Point	3	261	25.9	< 0.0001
Group*Treatment Centre	1	261	10.1	0.0016
Group*Time Point*Treatment Centre	6	261	6.4	**< 0.0001**

There was an interaction between treatment group, time-point and treatment centre (p < 0.0001). Therefore any difference in OHIP-14 summary scores between groups over time was not the same in the two centres. The group effect, time-point effect, centre effect and any of their two-level interactions cannot be interpreted in isolation. The groups were compared at each of the 3 time-points within each treatment centre separately. This analyses illustrated that the SDA group had better OHIP-14 scores compared to the RPD group in SFDH by an average of 2.7 at 1 month (p = 0.0056), 4.4 at 6 months (p < 0.0001), 5.6 at 12 months (p < 0.0001) and 7.9 at 24 months (p < 0.0001). In CUDH the SDA and RPD groups recorded similar summary OHIP-14 scores at 1 month (p = 0.1824) and 6 months in (p = 0.1970). However, the SDA group had better OHIP-14 scores by an average of 1.4 at 12 months in CUDH (p = 0.0461) and 2.9 at 24 months in CUDH (p < 0.0001). The model indicated that there was no difference between social classes (p = 0.7923) or genders recorded (p = 0.4217).

### OHIP-14 domains

A mixed model analysis of covariance for repeated measures was fitted to the following OHIP-14 domains: Functional Limitation, Physical Pain, Psychological Discomfort, Physical Disability and Psychological Disability. Insufficient variability was present to allow further analysis of the following OHIP-14 domains: Social Disability and Handicap. As before, fixed factors in the model were treatment group, time point (1, 6, 12 or 24 months), treatment centre, social class and gender.

Within the model the covariates used were the baseline values, comorbidity scores and age. The two-level interactions between treatment group and each of time point, treatment centre, social class, gender, comorbidity scores and age were considered for inclusion. These were retained in the model only if significant at the 5% level of significance. Relevant three-level interactions based on the significant two-level interactions were also considered for inclusion. These were to be retained in the model only if significant at the 5% level of significance. The random effect used in the model was the patient with time-point as the repeated factor. The covariance structure applied was spatial power based on time-point. The model assumptions were checked using residual analyses. Square root transformations were required for Functional Limitation, Physical Disability and Psychological Disability to normalise and/or stabilise the residuals to ensure the assumptions underlying the statistical models were met. Differences in mean scores for statistically significant factors or interactions were estimated using appropriate contrasts within the analysis of covariance models.

#### Functional limitation

At 24 months the SDA group reported lower scores of functional limitation by an average of 0.2 (p = 0.0316) compared to the RPD group (Table 4). There were no significant differences recorded between the two treatment centres. For the RPD and SDA treatment groups, scores of functional limitation worsened slightly over time, with an average increase of 0.06 at 12 months (p = 0.0391) and a further increase of 0.2 at 24 months (p < 0.0001).

**Table 4:** Functional limitation domain scores for each treatment group.

	Time-Point														
	0			1			6			12			24		
	n	mean	SD	n	mean	SD	n	mean	SD	n	mean	SD	n	mean	SD
**RPD**	65	1.3	1.4	53	0.6	0.8	45	0.4	0.7	45	0.5	0.8	44	0.9	0.9
**SDA**	67	1.3	1.4	52	0.4	0.8	47	0.5	0.9	47	0.5	0.9	45	0.6	0.8

#### Physical pain

Mean scores for physical pain are illustrated in Table 2. In SFDH the SDA group reported lower physical pain scores by an average of 0.9 at 1 month (p = 0.0442), 2.4 at 6 months (p < 0.0001), 2.4 at 12 months (p < 0.0001), and 2.6 at 24 months (p < 0.0001). In CUDH the functional and conventional groups reported similar physical pain scores at 1 month, 6 months, and 12 months, however, the functional group had lower scores by an average of 0.7 at 24 months (p = 0.0316).

#### Psychological discomfort

Mean scores for psychological discomfort are illustrated in Table 2. In SFDH the SDA and RPD groups reported similar psychological discomfort scores at 1 month and 6 months, however, the functional group had lower scores by an average of 1.6 at 12 months (p < 0.0001), and 1.5 at 24 months (p = 0.0001). In CUDH the SDA and RPD groups reported similar psychological discomfort scores at 1 month and 6 months, however, the SDA group had lower scores by an average of 0.6 at 12 months (p < 0.0001), and 0.6 at 24 months (p = 0.0001).

#### Physical disability

The SDA group recorded lower physical disability scores by an average of 0.4 at 1 month (p = 0.0161), 0.4 at 6 months (p = 0.0280), 0.6 at 12 months (p = 0.0036), and 0.9 at 24 months (p = 0.0001). There was no significant differences recorded between the two treatment centres (Table 2).

#### Psychological disability

Mean scores for psychological disability are illustrated in Table 2. In SFDH the SDA and RPD groups reported similar psychological disability scores at 1 month, however, the SDA group had lower scores by an average of 0.6 at 6 months (p = 0.0283), 0.6 at 12 months (p = 0.0308), and 1.9 at 24 months (p < 0.0001). In CUDH the two treatment groups reported similar psychological disability scores at 1 month, 6 months, and 12 months, however, the SDA group had lower scores by an average of 0.5 at 24 months (p = 0.0047).

## Discussion

This study represents one of a very small number of clinical trials within clinical prosthodontics with a substantial follow up period. Furthermore, the use of a validated patient centred outcome, oral health-related quality of life, as the primary outcome measure is innovative in this field. It has been suggested that to promote patient-centred care, clinicians should measure the health status of their patients using standardised questionnaires and use this information to inform clinical decision making [14]. The oral health impact profile (OHIP) is a widely reported and validated tool used to capture oral health-related quality of life (OHRQoL) particularly amongst older adults [15,16].

The results of this study illustrate that treatment according to the SDA concept resulted in significantly better mean OHIP-14 scores compared with RPD treatment for this group of partially dentate older patients, two years after treatment intervention. These results provide a longer and more meaningful follow up period compared with those previously reported from this study [2]. The results collected were consistent across both treatment centres where the SDA group recorded better mean OHIP-14 scores at all time points during the study. The results collected illustrate that the initial improvement in mean OHIP-14 score after 1 and 6 months was maintained in the SDA group through to 24 months. In comparison, the initial improvements observed in the RPD group began to be reversed after 6 months, particularly for those patients treated in SFDH. Thus, the benefit of SDA treatment appears to increase over time, particularly for those patients treated in SFDH.

Locker conceptualised that oral disease leads to oral ill-health through a sequence of inter-related domains [17]. The pain and functional limitation associated with oral impairment results in physical, psychological, and social disability, which in turn leads to patient handicap. OHIP-14 assesses each of these domains separately using two questions (e.g. “Have you had trouble pronouncing any words because of problems with your teeth, mouth or dentures?”, as an assessment of functional limitation). Although in principle the reliability of an index falls with a reduction in the number of items, OHIP-14 has been shown to retain the original conceptual dimensions of the longer version OHIP-49 with good reliability, validity, and precision [11,18].

A number of factors may explain these differences between treatment groups in the two treatment centres. Those patients in CUDH were treated in a conventional clinical environment within a modern tertiary healthcare centre with all of the advantages associated with this setting. Conversely, SFDH did not represent a typical dental clinical environment as patients were treated in a dental chair with a mobile dental unit. The mobile clinic was set up on each occasion and was not a permanent fixture in the geriatric day hospital. When not in use to provide dental services, the clinic was used to house podiatry and physiotherapy services. The patients themselves were also different with those treated in SFDH representing an older, more systemically unwell cohort. Many of the patients treated in SFDH were recovering from major medical conditions and often required transportation via ambulance to the hospital. Constructing fixed and removable prostheses were challenging in this environment but whilst RBB was often fabricated from a single good quality impression, RPDs required a larger number of complex clinical procedures over a longer timeframe [7].

This study adds further weight to the argument that functionally orientated tooth replacement is an acceptable treatment strategy for partially dentate older patients. The results reported were obtained using a gold standard methodology with a relatively low dropout rate. Previous work has already demonstrated that this approach can also have some positive impacts on nutritional status for partially dentate older patients [19]. Furthermore, it has been demonstrated that functionally orientated care is more cost effective to deliver and maintain compared to RPDs in this patient group [7]. The cost effectiveness analysis illustrated that the maintenance burden of patients with RPDs was significantly higher than those treated with the SDA. RPD patients returned for follow-up care more than three times more frequently compared with those in the SDA group (p<0.001). Similar findings have been observed in other studies which have demonstrated increased maintenance burdens associated with RPDs compared to the SDA approach where patients have suffered from higher incidences of new carious lesions, periodontal breakdown and technical complications when treated with RPDs [20–23].

Given the increasing evidence in favour of functionally orientated tooth replacement for partially dentate older patients this may have policy implications for public and private healthcare providers. Currently many publically funded healthcare systems, including those in Ireland, do not financially support or remunerate the SDA treatment as described in this study. Treatment is currently focused on RPDs which may be increasingly inappropriate for this patient group. Further research is required to explore the external validity of the treatment approach described in this study, particularly in a primary care context.

## Conclusion

Patients in the SDA Group generally maintained their improvements in OHIP-14 scores throughout the 24 month study period. Patients in the RPD Group recorded initial improvements in OHrQOL but for many these began to diminish 6 months after treatment. The benefits of functionally orientated treatment appeared to increase over time, particularly for the older, more systemically unwell patient cohort treated in SFDH.

## Supporting information

S1 Table CONSORT 2010 Checklist. (DOC)

S1 Fig Study protocol. (DOC)

S2 Fig Application for ethical approval. (DOC)

## References

[1] Watt R, Steele J, Treasure E, White D, Pitts N, Murray J. Adults Dental Health Survey 2009; implications of findings for clinical practice and oral health policy. *British Dental Journal* 2013; 214: 71–75. 10.1038/sj.bdj.2013.50 23348457

[2] McKenna G, Allen P, O’Mahony D, Cronin M, DaMata C, Woods N. The impact of rehabilitation using removable partial dentures and functionally orientated treatment on oral health-related quality of life: a randomised controlled clinical trial. *Journal of Dentistry* 2015; 43: 66–71. 10.1016/j.jdent.2014.06.006 24973731

[3] Kayser A. Shortened dental arches and oral function. *Journal of Oral Rehabilitation* 1981; 8: 457–462. 6975361 10.1111/j.1365-2842.1981.tb00519.x

[4] Jepson N, Allen PF, Moynihan P, Kelly P, Thomason M. Patient satisfaction following restoration of shortened mandibular dental arches in a randomised controlled trial. *International Journal of Prosthodontics* 2003; 16: 409–14. 12956497

[5] Kanno T, Carlsson G. A review of the shortened dental arch concept focusing on the work by the Kayser / Nijmegen group. *Journal of Oral Rehabilitation* 2006; 33: 850–862. 10.1111/j.1365-2842.2006.01625.x 17002745

[6] McKenna G, Allen P, O’Mahony D, Flynn A, Cronin M, DaMata C et al Comparison of functionally orientated tooth replacement and removable partial dentures on the nutritional status of partially dentate older patients: a randomised controlled trial. *Journal of Dentistry* 2014; 42: 653–659. 10.1016/j.jdent.2014.03.005 24657555

[7] McKenna G, Allen F, Woods N, O’Mahony D, Cronin M, DaMata C et al Cost-effectiveness of tooth replacement strategies for partially dentate elderly: a randomised controlled clinical trial. *Community Dentistry and Oral Epidemiology* 2014; 42: 366–374. 10.1111/cdoe.12085 24251386

[8] Thomason JM, Moynihan PJ, Steen N, Jepson NJ. Time to survival for the restoration of the shortened lower dental arch. *Journal of Dental Research* 2007; 86: 646–650. 10.1177/154405910708600712 17586712

[9] Giuney H, McKenna G, Whelton H, O’Mullane D. Is the shortened dental arch an underused treatment strategy in the Republic of Ireland? *Community Dental Health* 2011; 28: 265–268. 22320063

[10] Adult Dental Health Survey 2009. Department of Health, London, United Kingdom, 2011.

[11] Slade GD. Derivation and validation of a short-form oral health impact profile. *Community Dentistry and Oral Epidemiology* 1997; 25: 284–290. 9332805 10.1111/j.1600-0528.1997.tb00941.x

[12] Salvi F, Miller MD, Grilli A, Giorgi R, Towers AL, Morichi V et al A manual of guidelines to score the modified cumulative illness rating scale and its validation in acute hospitalized elderly patients. *Journal of the American Geriatric Society* 2008; 56: 1926–1931. 10.1111/j.1532-5415.2008.01935.x 18811613

[13] General Register Office: Classification of Occupations. The Stationary Office, London, United Kingdom 1966.

[14] Rumsfeld JS. Health status and clinical practice: when will they meet? *Circulation* 2002; 106: 5–7. 12093759 10.1161/01.cir.0000020805.31531.48

[15] Slade GD, Sanders AE. The paradox of better subjective oral health in older age. *Journal of Dental Research* 2011; 90: 1279–1285.\ 10.1177/0022034511421931 21917599

[16] Masood M, Newton T, Bakri NN, Khalid T, Masood Y. The relationship between oral health and oral health related quality of life among elderly people in United Kingdom. *Journal of Dentistry* 2017; 56: 78–83. 10.1016/j.jdent.2016.11.002 27825838

[17] Locker D. Measuring oral health: a conceptual framework. *Community Dental Health* 1988; 5: 3–18. 3285972

[18] Nunnally JC. 1967 Psychometric theory. New York: McGraw-Hill 192–3.

[19] McKenna G, Allen PF O’Mahony D, Cronin M, DaMata C, Woods N. Impact of tooth replacement on the nutritional status of partially dentate elders. *Clinical Oral Investigations* 2015; 19: 1991–1998. 10.1007/s00784-015-1409-4 25644134

[20] Budtz-Jorgensen E, Isidor F. A 5-year longitudinal study of cantilevered fixed partial dentures compared with removable partial dentures in a geriatric population. The *Journal of Prosthetic Dentistry* 1990; 64: 42–47. 2200879 10.1016/0022-3913(90)90151-2

[21] Jepson NJ, Moynihan PJ, Kelly PJ, Watson GW, Thomason JM. Caries incidence following restoration of shortened lower dental arches in a randomized controlled clinical trial. *British Dental Journal* 2001; 11; 140–144 10.1038/sj.bdj.4801122 11523885

[22] Walter MH, Marre B, Vach K, Strub J, Mundt T, Stark K et al Management of shortened dental arches and periodontal health: 5-year results of a randomised trial. *Journal of Oral Rehabilitation* 2014; 41: 515–522. 10.1111/joor.12160 24673467

[23] Wolfart S, Marre B, Wostmann B, Kern M, Mundt T, Luthardt RG et al The randomised shortened dental arch study: 5-year maintenance. *Journal of Dental Research* 2012; 91: 65S–71S. 10.1177/0022034512447950 22699671

