# Supplementary material for: Impact of oral rehabilitation on the quality of life of partially dentate elders in a randomised controlled clinical trial: 2 year follow-up
Source: PLoS One. 2018 Oct 11;13(10):e0203349. doi: 10.1371/journal.pone.0203349 (PMC6181283; doi:10.1371/journal.pone.0203349)
Supplement: S1 Fig — (DOC) [file pone.0203349.s002.doc]

##

## October 2007

**Research Proposal Outline**

**Determining the impact of oral status on health of older Irish adults**

**Dr. Finbarr Allen, School of Dentistry, UCC.**

**Dr. Denis O’Mahony, Dept. of Medicine, UCC.**

**Dr. Michael Cronin, Dept of Statistics, UCC.**

**Dr. Gerry McKenna, School of Dentistry, UCC.**

# Current Knowledge

*Present status of oral health of older adults*

Currently available evidence from population surveys indicates that the proportion of adults over the age of 60 years is expanding rapidly across the European Union countries, including the Republic of Ireland.^1^ This trend mirrors the arrival of the “baby boomer” generation into early old age. As the population ages, dental health surveys indicate that the proportion of older adults retaining natural teeth into old age is increasing.^2,3^ The trend of increasing tooth retention (as opposed to total toothloss, which has been steadily decreasing over the past 30 years) has been welcomed as a sign of improving dental health. However, chronic dental and oral diseases such as dental caries and periodontal disease continue to be a significant health problem among older adults.

The natural history of pathological processes affecting teeth indicates that the effects of these diseases are progressive and cumulative. As stated by Petersson^4^, this rapidly changing global disease pattern is closely linked to changing lifestyles, which include diets rich in sugars, widespread use of tobacco and increased consumption of alcohol. These lifestyle factors also significantly impact on oral health, and oral diseases qualify as major public health problems owing to their high prevalence and incidence in all regions of the world. Like all diseases, they affect primarily the disadvantaged and socially marginalised populations, causing variable degrees of pain and suffering, impairing function and impacting on quality of life. Traditional treatment of oral diseases is extremely costly even in industrialised countries and is unaffordable in many cases. Accordingly, a major challenge for the dental profession will be to plan oral healthcare for older adults which is affordable, readily accessible and positively impacts upon their quality of life.

Some researchers have suggested that older adults have different functional needs to young patients and therefore do not need a complete natural dentition.^5^ Furthermore, the WHO suggested that a goal for oral health in the year 2000 should be that adults retain for life a healthy, functioning dentition of at least twenty teeth and not require an oral prosthesis to replace missing teeth.^6^ The concept of “minimally invasive dentistry (MID)” has also been proposed as an effective and acceptable form of dental caries management for older adults.^7,8^

These functionally oriented treatment strategies aim to reduce the burden of maintenance for older adults, and have been advocated as a means of providing a natural, functional dentition.

In a recent publication, the World Health Organisation has highlighted the paucity of research into the oral health needs of older adults.^9^ Building and strengthening research capacity in public health are highly recommended by WHO for effective control of disease and the socioeconomic development of any given country. The WHO Oral Health Programme encourages public health care administrators and decision makers to design effective and affordable strategies for better oral health and quality of life of older adults, which in turn, are integrated into general health management programmes. This has yet to occur in the Republic of Ireland.

*Problems associated with poor oral health status – nutrition considerations*

Poor dentition and oral health often have very significant negative effects on dietary intake and nutritional status and consequently on overall functional status and quality of life in the older adult. In a recent position paper, the American Dietetic Association^10^ stated that “Oral health and nutrition have a synergistic bidirectional relationship. Oral infectious diseases, as well as acute, chronic, and terminal systemic diseases with oral manifestations, impact the functional ability to eat as well as diet and nutrition status. Likewise, nutrition and diet may affect the development and integrity of the oral cavity as well as the progression of oral diseases.”

The loss of natural teeth is related to diminished nutritional intake, especially in older adults. In studies of nutrition in adult populations, poor quality diets have been reported in adults missing natural teeth and wearing partial and complete dentures.^11-13^ The reasons for this are thought to be difficulty in chewing hard foods such as raw vegetables and fruit and decreased sense of taste. Conversely, there is some limited evidence that improvement of dentition and oral health generally has very positive effects on these parameters. In the recent U.K. National Diet and Nutrition Survey of people aged 65 years and over, Steele et al^12^ reported on the oral health of the participants in the survey. A consistent finding in their report was that dentate individuals had higher daily intake of protein, fibre, calcium, iron and vitamin C than their edentulous counterparts. This has implications for general health in adults, as poor diet may lead to deficiency of nutrients and illnesses such as osteoporosis, atherosclerosis and bowel disease. Although nutritional state is influenced by factors such as age, socio-economic status and general health, it would appear that dental status is an important co-factor. Poor nutritional status is apparent in elderly edentulous adults, particularly those living in institutions. Although there are many factors which influence food selection, it seems likely that preservation of a critical number of natural, disease free teeth is a significant factor facilitating a healthy diet. These UK findings have been confirmed in other studies of frail elders including Finland^14^ and Brazil.^15^ The risk of malnutrition in frail elders is such that Poulsen and co-workers^16^ have recently recommended oral examination should be part of routine hospital admission procedures for geriatric admissions.

Our studies in Cork have shown that edentulous patients are at moderate risk of poor nutrition status. In one of these studies^17^, 35 independently living edentulous patients (23 female, 12 male) ranged in age from 52-77 years (median 65 years) agreed to take part in the study of the impact of new dentures on diet and quality of life. Prior to treatment, all subjects completed a questionnaire which contained a number of validated social resources, oral health related quality of life and the short form of the Mini Nutritional Assessment (MNA, described later). Three quarters of the sample felt they had no nutritional problems. However, approximately 70% reported that they had changed their food choices because of dental problems. The mean MNA score pre-treatment was 6.23 (±1.48), which would be in the medium risk range. The post-treatment MNA score remained very similar to the pre-treatment score, suggesting that provision of new complete dentures had not altered food consumption behaviour.

In a separate study^18^, we assessed the nutrition status of partially dentate adults (including adults wearing partial dentures) and found that the mean short form MNA score was 8.41(±1.04). This suggests that partially dentate adults are at a lower risk of poor nutrition status than edentulous adults. In partially dentate patients, the contribution of removable partial dentures to masticatory ability is questioned. There is some evidence that masticatory ability is not enhanced by partial dentures once when the patient has a minimum of twenty teeth.^19^ This may be of importance, as partial dentures in older adults are associated with higher disease prevalence. If there is minimal gain in terms of function, then the biological price of wearing a denture may not be worth paying in older adults. This requires further research to improve knowledge in this area, particularly in patients at risk of poor nutrition.

*Problems associated with poor oral health status – general health status*

Increasingly, it is accepted that there is an interrelationship between oral health and general health in older people. It is apparent that oral diseases have risk factors in common with other chronic diseases. Systemic diseases and/or the adverse side effects of their treatment can lead to increased risk of oral disease, reduced salivary flow and loss of oral comfort. In addition to the impact of systemic disease on oral health, there is a burgeoning interest in putative associations between oral disease, particularly inflammation of the periodontal tissues surrounding natural teeth (“Periodontitis”), and Coronary Artery Disease (CAD), Diabetes Mellitus and Respiratory Disease. Given that there is certainly a unidirectional, and possibly a bidirectional, link between oral and general health, the benefits of a disease free mouth to an older adult are considerable.

*Problems associated with poor oral health status – quality of life*

As populations age and lose teeth, their quality of life may be expected to change, particularly with respect to the way that their oral condition impacts on day to day activities. This may happen as a direct result of altered function due to tooth loss, but possibly also as a result of changes in perceptions and values that occur with increasing age. A number of other factors may modify this process, for example the social and cultural norms to which populations are exposed. Quality of life is affected in some way by oral health in the majority of people.^20^ Understanding the relationship between age- related, dental and cultural influences on quality of life has relevance if we wish to measure oral health inequalities within and between populations. A recently published study used nationally representative population datasets to explore the relationship between age, tooth loss and oral health related quality of life.^21^ They reported that age and tooth loss are closely associated, but have independent effects on oral health related quality of life. Tooth loss (which is associated with increasing age) is associated with more negative impacts, whilst increasing age independently results in fewer. In all of the populations and sub-populations studied, a complete or almost complete natural dentition was associated with the best oral health related quality of life.

*The situation in the South of Ireland*

At the present time, very little is known about the oral health behaviours of older adults in the Republic of Ireland. In addition to sporadic uptake of dental care at present, there are also inequalities in the funding of dental care in Ireland. A significant portion of the budget (approximately 50%) for publicly funded dentistry is currently allocated to adults in employment. Older adults who are passed retirement age have a far more limited access to dental schemes supported by public funding. Dental care can be accessed through the DTSS scheme for holders of a Medical Card, and this is automatic for adults over 70 years. It appears that uptake of dental care schemes nationally is sporadic, with approximately 30% of adults attending routinely for dental care (personal communication, Dept. of Health & Children). This phenomenon may relate to anxiety about dental treatment and associated financial costs, or, difficulties accessing dental care. Accordingly, older adults tend to have higher disease prevalence.^2,3^ The recently published survey of oral health of Irish adults indicates that periodontal disease and pathology of the oral mucosa are more prevalent in adults over 65 years than younger cohorts in the survey. This should be addressed in order to broaden the range of oral healthcare possibilities for older adults in the future.

In the past, oral healthcare for older adults was dominated by tooth extraction and provision of dentures, and this may no longer be acceptable to older adults. A number of studies in the UK and the Netherlands have suggested that partially dentate adults are not happy with the prospect of wearing dentures to replace missing teeth.^22-23^ Compliance with wearing partial dentures has proved to be variable, with non-wearing of dentures reported to be as high as 40% in some studies.^24^ Accordingly, there is a need for research to further determine the type of care most acceptable to older adults

The following proposal is underpinned by the recommendation of the World Health Organisation that “global strengthening of public health programmes through implementation of effective oral disease prevention measures and health promotion is urgently needed, and common risk factors approaches should be used to integrate oral health with national health programmes”.^9^ The proposal is also based on the premise that oral healthcare programmes for older adults must address their specific needs, recognise diversity in demand, be readily accessible, and, affordable for public fund providers.

**Overall** **Aims and Objectives of Research Project**

- To assess the impact of dental status on oral health related quality of life of Irish older adults.
- To assess the impact of dental status on nutrition status of Irish older adults.
- To undertake a clinical trial of the effect of a targeted therapeutic intervention on oral health related quality of life and nutrition status of older adults.

**Target Population**

Our target population will be Irish adults over the age of 65 years living in Cork. We will recruit participants from two centres:

1. Patients attending the Geriatric Day Hospital facility at St. Finbarr’s Hospital, Cork. These are mostly over the age of 75 years, and may be considered “at risk” of developing nutritional problems associated with tooth-loss.

2. Patients attending for dental assessment and/or treatment at Cork University Dental School & Hospital. These patients may not pose the same level of risk for nutritional problems as those attending the Geriatric Day Hospital.

Our rationale for using these two groups is to target a broad range of “at risk” patients. This would include those with primarily medical problems and those with primarily dental problems.

**Stage 1: To assess the impact of dental status on oral health related quality of life and nutrition status of Irish older adults.**

The **aims** of this part of the study are:

1) To identify older patients “at risk” of medical complications associated with poor oral health. The screening procedure will involve the use of existing objective measures of disease and validated subjective health status measures.

2) To determine if the threshold of 10 retained natural teeth per jaw with 3-5 occluding (biting) contacts is clinically relevant in terms of nutrition status and health related quality of life.

The **objective** of this part of the study is to show that retention of a functional dentition which provides 3-5 occluding contacts has a positive impact on nutrition status and health related quality of life.

**Stage 1: Methods**

We will recruit patients for this aspect of the study from both centres. The objective indicators of oral disease will be a basic periodontal examination (BPE), the Decayed, Missing and Filled Teeth (DMFT) index and, the number of sound and untreated teeth (SUNT). We will also record details of the number of occluding contacts (tooth/tooth; tooth/denture; tooth/bridge pontic) and design and type of prosthesis already present. These screening measures are widely used in population surveys of adult oral health.

Subjective assessment of **oral health related quality of life** will be made using validated health status measures. The short form of the Oral Health Impact Profile (OHIP-14).^25^ has been validated for use in older adults and reported in the literature, and the PI has used the measure in clinical trials.^26^ The OHIP is a comprehensive measure of oral health which contains seven conceptual domains, namely, functional limitation, pain, psychological discomfort, physical disability, psychological disability, social disability and handicap. The short form of the OHIP contains 14 items, and the response format of the questions is “During the past year, have you had …. because of problems with your teeth, mouth or dentures? Responses to the statements are based on the Likert scale (i.e., 0=never, 1=hardly ever, 2=occasionally, 3=fairly often and 4=very often). Summary scores are calculated by summing response codes.

We will assess **nutrition status** using the full Mini Nutritional Assessment (MNA)^27^, which includes measurement of Body Mass Index (BMI). This measure also includes subjective assessment and dietetic components and has been widely used as an outcome measure in studies of older adults, including our studies in Cork.^17,18^  The MNA involves subjective assessment of appetite, mobility, dietary behaviours in addition to measurement of Body Mass Index. Using the MNA scoring system, if a patient scores <17 points or below out of a possible 30 points, this indicates that the patient is malnourished. A score of 17-23.5 points indicates that the patient is “at risk” of malnutrition.

Following blood sample collection, a general health assessment of nutritional status will be made by measuring:

- C Reactive Protein/serum albumin ratio
- serum cholesterol
- total lymphocyte count
- Ferritin
- Folate
- Vit B12
- Vit D

Norms for these parameters are available, and along with the MNA score, these data will indicate the level of malnutrition risk for each patient.

In recording these objective and subjective measures, we aim to determine whether there is a “threshold” in terms of numbers of retained natural teeth which is correlated with a) biological markers of nutrition status, and, b) health related quality of life status. The World Health Organisation (WHO) has suggested that functionally orientated treatment planning should aim to preserve 20 natural teeth for older adults.^6^ Many older adults will have fewer than 20 natural teeth, but it may be feasible to provide a shortened dental arch in one jaw. Therefore, in addition to the number of retained teeth, the distribution and number of occluding pairs/contacts is also important. The **hypothesis** that we will test, therefore, is that there are clinically relevant differences in nutritional status and quality of life in patients with ≥ 3-5 pairs of contacting teeth (tooth/tooth and/or tooth/denture tooth contacts) compared with patients who have <3 pairs of contacting teeth (tooth/tooth and/or tooth/denture tooth contacts. Patients recruited in this stage of the study will also be invited to participate in Stage 2 described next.

**Stage 2: To undertake a clinical trial of the effect of a targeted therapeutic intervention on oral health related quality of life and nutrition status of older adults**.

This population is unlikely to have a disease free natural dentition. It will be important to devise a treatment protocol which is aimed at preserving a functional natural dentition in older adults. Many older adults are reluctant to accept complex treatment forms of treatment, and may prefer to have simple forms of treatment aimed at providing a functional rather than complete dentition. However, the impact of functionally orientated treatment versus conventional treatment has not been compared in terms of its impact on nutrition status and oral health related quality of life. In relation to quality of life, the concept of *minimally important clinical difference* has been described by Jaeschke et al^28^ as “The smallest difference in score in the domain of interest which patients perceive as beneficial and which would mandate, in the absence of troublesome side-effects and excessive cost, a change in the patient’s management”. This suggests that if an intervention does not produce such a change in quality of life score, then it is not worth providing and unlikely to be perceived as beneficial to patients. If two forms of treatment produce minimally important clinical differences, then logic dictates that the more conservative of these treatments is to be recommended. A study reported by Locker et al^29^ has determined that a change of 5 on the OHIP-14 scale is the minimally important clinical difference for this scale.

The **aim** of this phase of the study is to compare the outcome of two treatment strategies on these two variables. We will test the following **hypotheses:**

1. That oral health related quality of life in elderly patients managed by functionally oriented treatment planning is no worse than those managed by conventional treatment.
2. That nutrition status in elderly patients managed by functionally oriented treatment planning is no worse than those managed by conventional treatment.

**Stage 2: Patients & Methodology**

We will recruit consecutive patients for a prospective randomised clinical trial from the two study centres, ie, those currently attending the Geriatric Day Hospital and, the Dental School & Hospital. The inclusion criteria are the following:

1. A minimum of six natural teeth in at least one jaw.

2. No medical complications which contraindicate routine dental treatment (e.g., unstable Angina; INR level > 4; high risk of Infective Endocarditis)

3. No evidence of Dementia

4. Able to have dental treatment in a dental chair

5. Able to communicate in English

***Randomisation strategy:***

Patients that are willing to be treated and who satisfy the inclusion criteria will be randomly allocated to one of two treatment groups: Group 1 will be managed using functionally oriented treatment planning; Group 2 will receive conventional treatment aimed at restoring a complete dentition. Randomization will be performed using a computer generated schedule in SAS®. Randomization will be in blocks of varying length and will be stratified according to gender. Separate randomization schedules will be generated for both recruitment sites. It is anticipated that the group at St. Finbarr’s Hospital will have a higher prevalence of co-morbidity. This will be assessed pre-treatment using the Barthel Index to indicate functional status. We will calculate a Barthel Index score for all patients at both sites and the randomization schedule will be further stratified according to this score.

Patients will be advised that they will receive treatment based upon random assignment to one of the treatment arms. If they do not to consent to treatment, but agree to provide blood samples and complete the questionnaires, we will consider them an “untreated control” group for comparison purposes. For comparison purposes, we will also collect data from patients with no natural teeth (i.e., edentulous). It is anticipated that there will be a significant refusal rate (possibly as much as 50%) for treatment, particularly in the older cohort of patients at St. Finbarr’s Hospital. Our power calculation to determine sample size has considered this possibility.

Treatment will be provided at the two centres, Cork University Dental School & Hospital, and, St. Finbarr’s Hospital. Dental screening/examination and treatment required to manage dental caries, restore teeth and provide dentures will be undertaken by a PhD student who will be a trained Dentist, registered with the Dental Council of Ireland. Oral hygiene instruction and non-surgical periodontal therapy will be provided by a trained Dental Hygienist.

A screening history, checklist of adverse dentition/oral health risk factors and a systematic dental/oral examination will be performed in each patient prior to treatment. Caries status will be recorded using the DMFT and Root Caries indices (RCI). For the DMFT, details of diseased, filled and missing teeth will be recorded separately. Periodontal disease status will be measured by recording periodontal pockets around natural teeth (6 measurements per tooth) and gingival bleeding scores. Details of type and design of dentures (complete and partial), if present, will be recorded. Finally, a proforma for screening medical history used in national surveys of oral health will also be completed at baseline.

Those patients who have evidence of dental pathology and are missing natural teeth will be offered entry into the clinical trial. These patients will be randomly allocated into one of the following treatment arms:

***Group 1***: ***Functionally oriented treatment planning including minimally invasive dentistry:***

Following an initial clinical examination by a Dentist, a functionally oriented treatment plan with minimal intervention will be implemented. Teeth deemed to have a hopeless prognosis (where affected teeth are deemed unrestorable, or, grade 3 mobility is evident) will be extracted. The following intervention will then be provided:

- Oral Hygiene advice will be provided using a standardised protocol
- Non-surgical periodontal treatment will be provided for management of periodontal disease
- Atraumatic Restorative Treatment (ART) using hand excavators to remove diseased dental tissue and adhesive restorative materials to restore cavities, will be provided for management of dental caries (both coronal and root caries where present).
- Restoration of a shortened dental arch in both jaws (or one jaw if the patient is edentate in the upper or lower jaw) to provide 3-5 occluding pairs of natural and replacement teeth using simple adhesive procedures (either with fibre reinforced composite resin impregnated strips, or, resin bonded cast metal bridges using a Maryland design)

***Group 2***: ***Restoration of complete dental arch/arches with conventional intervention***

Following an initial clinical examination by a Dentist, a treatment plan aimed at restoring a complete dentition will be implemented. Teeth deemed to have a hopeless prognosis (where affected teeth are deemed unrestorable, or, grade 3 mobility is evident) will be extracted. The following intervention will then be provided:

- Oral Hygiene advice will be provided using a standardised protocol
- Non-surgical periodontal treatment will be provided for management of periodontal disease
- Conventional caries management with rotary instruments and restoration with plastic (resin based or amalgam) restorative materials.
- Restoration of a complete dental arch comprising natural teeth and using removable partial dentures to replace missing teeth.

In both arms of the trial, the intention is to render patients pathology free and, therefore, dentally fit.

***Data Collection & Outcome Measures***:

Data will be collected at three time points, namely: 1) pre-treatment; 2) 3 months, and; 12 months following completion of treatment. The **primary** outcome of interest in this part of the study will be oral health related quality of life as indicated by the short form Oral Health Impact Profile (OHIP). The **secondary** outcome of interest is nutrition status as determined by the Mini Nutritional Assessment (MNA).

We aim to assess if there are benefits, and if these benefits are short term or maintained in the medium term.

Follow-up data collection will include a dental examination to check for the incidence of new pathology, collection of a blood sample (screen for C Reactive Protein/Albumin ratio, Lymphocyte count, serum cholesterol, Ferritin, Folate, Vit B12 and Vit D) and, completion of OHIP-14 and MNA questionnaires.

**Statistical Analysis & Power Calculation**

The power calculations have been made on summary OHIP-14 score data available from the UK Adult Dental Health Survey and on summary MNA data collected in studies performed in Cork. In both cases the null hypothesis is that patients receiving conventional treatment (Group 1) have a better outcome than those managed using functionally oriented treatment planning (Group 2). The alternative hypotheses are that Group 1 is no better than Group 2. Power was set at 80% with a one-sided 5% level of significance.

For OHIP-14, the standard deviation was 7.4 and the maximum difference allowed between groups is 4.

For MNA, the standard deviation was 1.48 and the maximum difference allowed between groups is 0.80.

Based on these specifications, we need to recruit 44 patients to both treatment groups. To allow for a drop-out rate of up to approximately 30% (which may be pessimistic) during the study, we aim to recruit 65 patients per treatment group.

These sample sizes have been calculated to meet the objectives of stage 2. The sample size at stage 1 will be greater as those patients who subsequently refuse to be randomized and those that fail the inclusion criteria will still be included. Thus the sample size at stage 1 will be more than adequate to provide reliable estimates of OHIP-14 and MNA and to determine the effects of dental status on these.

All variables recorded at stage 1 will be summarized using appropriate descriptive statistics and graphics. Relationships between dental status (BPE, DMFT and SUNT) and both nutritional status (MNA, CRP/Albumin, Cholesterol, Lymphocyte count, Ferritin, Folate, Vit B12 and Vit D) and quality of life (OHIP-14) will be assessed using linear models and logistic regression (binary and ordinal) models. The threshold of retained natural teeth and occluding contacts will also be assessed using such models. Patient demographic variables (including age, gender, recruitment site and Barthel Index) will controlled for by including them in these models as covariates/factors.

All variables recorded at stage 2 will be summarized using appropriate descriptive statistics and graphics. These will be presented by time-point and by treatment group. Post-treatment assessments of nutritional status (MNA, CRP/Albumin, Cholesterol, Lymphocyte count, Ferritin, Folate, Vit B12 and Vit D) and quality of life (OHIP-14) will be compared between the two treatment groups using linear models and/or generalised linear models. Pre-treatment assessments will be included in these models as covariates and all comparisons will be controlled for patient demographic variables by including them in these models as covariates/factors. Comparisons of the two treated groups (individually and combined) to the untreated group will also be made.

All statistical analyses will be performed in SAS®.

**Project management information:**

**Key milestones/Timeframe:**

0- 12 months: Phase 1: Commence data collection using questionnaires and blood samples. Data entered; data analysed. Commence recruitment for Phase 2.

12-30 months: Phase 2: Clinical trial commenced; Treatment completed and pre-treatment data collected for all patients by month 18; Two follow up data collection points completed for all patients by month 30.

31-36 months: Report writing, preparation of PhD thesis, submission of final report to HRB

**Justification for Support Requested**

The nature of this study is such that a full-time researcher is required to manage the project. The originality of the research proposal makes it suitable for a PhD student (who needs to be a trained dentist), who will be mentored and supervised by the applicants. This individual will undertake the clinical work, and will organise for blood sample collections and administration of questionnaires at both centres. In order to undertake treatment at St. Finbarr’s, he/she will need a portable dental treatment kit. Oral hygiene instruction and basic tooth cleaning (“scaling”) procedures will be undertaken by a trained Dental Hygienist. A dental nurse will also be required to assist the Dentist at St. Finbarr’s. An administrative assistant is required to prepare questionnaires, organise appointments for the patients and to arrange postage of questionnaires when required. Some funding is required for a portable laptop computed on which data will be stored and analysed. Data from questionnaires will be cleaned and entered into a datafile by a Data Entry service.

**Implications for Research Findings**

Very little is currently known about the relationship between oral health status, nutrition status and oral health related quality of life of older Irish adults. In addition to significant knowledge gaps, access to oral healthcare for patients over the age of 65 seems to be limited. This study will indicate the significance of the influence of natural tooth retention on nutrition status and health related quality of life. We will also determine if functionally orientated treatment intervention has a positive impact on these variables. If such a relationship is demonstrated, then the findings of the study can be used to inform public health policy on oral healthcare delivery for elderly Irish adults, as recommended by the World Health Organisation.

**Key: 19848w19848n19850v19852v19894l49**
