# Supplementary material for: Impact of oral rehabilitation on the quality of life of partially dentate elders in a randomised controlled clinical trial: 2 year follow-up
Source: PLoS One. 2018 Oct 11;13(10):e0203349. doi: 10.1371/journal.pone.0203349 (PMC6181283; doi:10.1371/journal.pone.0203349)
Supplement: S2 Fig — (DOC) [file pone.0203349.s003.doc]

**UNIVERSITY COLLEGE CORK**

**Clinical Research Ethics Committee of the Cork Teaching Hospitals**

**PROTOCOL SUBMISSION FORM**

For New Protocols and Amendments to Existing Protocols Involving the Use of Human Subjects in Research

All items must be completed as indicated; incomplete applications will be returned. A protocol application must include:

1. Protocol Submission Form (pages 1 - 4) - **Original and one copy. Original must be signed on page four.**
2. Consent Form (the standard Ethics Committee format) - **Seven copies.**
3. Detailed Protocol including instruments involved, Investigators Brochure, IND, if applicable - **Seven copies.**
4. Details of insurance policies in place to cover the study.

To obtain **expedited review** or **waiver of consent,** please see page four of this application. For **exempt research,** refer to the Ethics Committee Policies and Procedures Manual (see below). For **amendments** to already existing protocols complete page one, signatures on page four and those sections of this form in which a change has occurred; all items left blank will indicate that there is no change in status. Explain exactly what you are changing from your original submission and the rationale for the change. If applicable, submit a revised protocol or consent form highlighting the changes from the original.

The complete application package must be received in the Ethics Committee office by the appropriate deadline in order to ensure review the next month. The Ethics Committee office is located at the Clinical Sciences Building, Cork University Hospital, Wilton, Cork. The telephone number is (021) 901255 / 345599 and fax number is (021) 343211.

**Investigator: Dr Gerald McKenna** **Appointment: Clinical Fellow in Restorative Dentistry**

Investigator must be a member of the faculty or a consultant.

**Office Address: Cork University Dental Hospital and School, Wilton, Cork** **Phone: 021 420 5033**

**Department:** Restorative Dentistry

**Title of Protocol:** Determining the impact of oral status on health and quality of life of older Irish adults.

**Co-Investigators:** Dr Finbarr Allen, Head of School and Consultant in Restorative Dentistry, School of Dentistry, UCC. Dr Denis O’Mahony, Consultant Geriatrician, Department of Medicine, UCC. Dr Michael Cronin, Department of Statistics, UCC.

Only the co-investigators listed may perform the procedures indicated on this protocol. They may NOT amend the protocol.

**Site of Performance of study: Cork University Dental Hospital and St Finbarr’s Hospital, Cork.**

**Status of Protocol:** **New ✓** **Amendment** If amendment, **Original Protocol #:_________**

**This protocol is part of an active or pending externally funded project: Yes ✓ No**

**If yes, complete the rest of the page:**

**Agency:** Health Research Board

**PI for grant or contract** (if not same as investigator responsible for this protocol): Dr Finbarr Allen

**Grant Proposal Title: Determining the impact of oral status on health and quality of life of older Irish adults**

**Please indicate if the Principal Investigator personally gains financially from this study Yes No ✓**

**Please indicate if there are any additional cost implications for hospital management beyond standard of care
Yes No ✓ N/A If yes, please specify**

To assist you in the submission process, the following items are available from the Ethics Committee office:

- Submission Deadline Schedule
- Sample Ethics Committee Consent Form and Consent Form Checklist
- Ethics Committee Policies and Procedures Manual
- Additional copies of this application
- The Manual includes such items as details of protocol construction, elements of informed consent, consent and assent from children, and protocols exempt from review.

Ethics Committee Protocol Submission Page Two

**SPECIAL CONSIDERATIONS** All items on this page must be completed.

1. This study is part of a **multi-centre** project **Yes No ✓**

If yes, identify:

1. This study involves the use of an **Investigational New Drug (IND): Yes No ✓**

**If yes, complete 2(a):**

(a) Drug Name, IND#, Company:

_______Phase I _______Phase II ________Phase III

1. This study involves **laboratory/clinical procedures** NOT part of ordinary management: **Yes No ✓**
2. This study involves the clinical experimental use of **radiation or radioisotopes:** **Yes No ✓**
3. This study involves the use of **biohazardous** or **infectious materials:** **Yes No ✓**

If yes, please explain:

1. Human subjects from the following **special population(s)** may be involved in this study:

___Infants (< 1 year) ___Children (1 - 17 years) **✓**_Elderly (> 59 years)
 ___Pregnant women ___Prisoners ___Mentally disabled
 ___Mentally retarded ___None of these

**No investigator shall recruit from a student group where he/she, or any of the co-investigators, have material influence over the assessment of academic performance of that student group.**

**PRECODED STUDY DESCRIPTION** For each of the categories below, please select the item(s) which best describe(s) your study. You may check up to two items for each category.

1. **Type of Study:** A)_ **✓** Behavioural-Social B)___Compassionate C)_ _Descriptive D)___Diagnostic E)___Educational F)___Epidemiologic G)___Preventive H)_ **✓**_Therapeutic
   Z) __Other:
2. **Organ System(s):** A)_**✓**_Not Applicable B)___Breast C)___Cardiovascular D)___Dermatologic E)___Endocrine/Metabolic F)___Gastrointestinal/Hepatic G)___Haematologic H)___Musculo-skeletal I)___Neurologic J)___Opthalmologic K)___Otolaryngolic L)___Pulmonary M)___Renal N)___Reproductive O)___Urinary Tract P)___Multisystem (>2 systems)
   Q)___Cells, blood, other body fluids or tissues only
   Z) __Other:
3. **Type of Disorder:** A) __Not Applicable B)___Congenital C) _Degenerative D)___Infectious E)___Immunologic/Inflammatory F)___Malignant G)___Metabolic/Endocrine
   H)_ Normal Physiologic I)___Psychiatric J)___Traumatic Z)__ **✓**_Other: Oral diseases__________________________________________________
4. **Type of Drug/Device:** A)_**✓**_Not Applicable B)___Analgesics C)___Anaesthetics
   D)___Anti-asthma/allergy E)___Anticoagulant F)___Antiinfectives G)___Antiinflammatory/Anticonvulsants H)___Biologicals/Vaccines I)___Blood Components J)___Cardiovascular/Antihypertensive K)___Chemotherapeutic Agents L)___Contraceptives M)___Contrast Media N)___Dermatologics O)___Diagnostics P)___Hormones Q)___Immunosuppressives R)___Sedatives/Antidepressants/Tranquilizers S)___Vitamins
   Z)___Other:__________________________________________________________________

Ethics Committee Protocol Submission Page Three

**PROTOCOL ABSTRACT** - (This page **must** be completed; Use additional pages as needed).

**Purpose of Investigation:** The aims of this investigation are:

To assess oral health behaviours of Irish older(> 65 years) adults.

To assess the impact of dental status on oral health related quality of life of Irish older adults.

To assess the impact of dental status on nutrition status of Irish older adults.

To undertake a clinical trial of the effect of a targeted therapeutic intervention on oral health related quality of life and nutrition status of older adults.

**Procedures to which humans will be subjected:**

Participants will undergo a standardised dental screening examination which will comprise a Basic Periodontal Examination ( BPE ) and teeth charting including the presence of dental caries, restored and missing teeth. We will also record details of the number of occluding contacts (tooth/tooth; tooth/denture; tooth/bridge pontic) and design and type of prosthesis already present.

Subjects will be asked to complete the short form of the Oral Health Impact profile ( OHIP-14 ) in order to assess oral health related quality of life.

Participants’ nutritional status will also be assessed using the Mini Nutritional Assessment ( MNA ) which includes measurement of Body Mass Index ( BMI ).

For those patients already undergoing routine haematological screening, a general health assessment of nutritional status will be made by measuring: C Reactive Protein/serum albumin ratio, serum cholesterol, total lymphocyte count, ferritin, folate, Vitamin B12 and Vitamin D.

**Potential benefits to subjects and/or society:** At the present time, very little is known about the oral health behaviours of older adults in the Republic of Ireland. It appears that uptake of dental care schemes nationally is sporadic, with approximately 30% of adults attending routinely for dental care (personal communication, Dept. of Health & Children). As yet, we do not know why attendance should be so sporadic, and a variety of reasons have been advanced for this. This phenomenon may relate to anxiety about dental treatment and associated financial costs, or, difficulties accessing dental care. In the past, oral healthcare for older adults was dominated by tooth extraction and provision of dentures, and this may no longer be acceptable to older adults. A number of studies in the USA, the UK and the Netherlands have suggested that partially dentate adults are not happy with the prospect of wearing dentures to replace missing teeth. Compliance with wearing partial dentures has proved to be variable, with non-wearing of dentures reported to be as high as 40% in some studies. Accordingly, there is a need for research to further determine the type of care most acceptable to older adults. In addition to sporadic uptake of dental care at present, there are also inequalities in the funding of dental care in Ireland. A significant portion of the budget for publicly funded dentistry is currently allocated to adults who are likely to have benefited from water fluoridation (< 40 years of age). This should be addressed in order to broaden the range of oral healthcare possibilities for older adults in the future. Dental care can be expensive for older adults, and, in the future, some dental procedures may be undertaken by trained dental auxiliaries as a means of reducing financial costs. Once again, the feasibility of this has not been explored in Ireland.

In relation to concerns about the impact of dental status on nutritional status, there are no reliable research data on whether a systematic, large-scale screening programme for at-risk older people has a large-scale positive effect on nutritional status and oral health related quality of life. More research is required to assess this, and to inform oral healthcare policy for older adults at a national level in Ireland.

The elderly population targeted in this study is unlikely to have a disease free natural dentition. It will be important to devise a treatment protocol which is aimed at preserving a functional natural dentition in older adults. Many older adults are reluctant to accept complex treatment forms of treatment, and may prefer to have simple forms of treatment aimed at providing a functional rather than complete dentition. However, the impact of functionally orientated treatment versus conventional treatment has not been compared in terms of its impact on nutrition status and oral health related quality of life.

This research proposal is underpinned by the recommendation of the World Health Organisation that “global strengthening of public health programmes through implementation of effective oral disease prevention measures and health promotion is urgently needed, and common risk factors approaches should be used to integrate oral health with national health programmes”. The proposal is also based on the premise that oral healthcare programmes for older adults must address their specific needs, recognise diversity in demand, be readily accessible, and, affordable for public fund providers.

**Potential risks to subjects and precautions taken to minimise risk:** All of the interventions proposed form part of recognised screening procedures. Therefore, patients should not require additional precautions due to participation in this study.

Patients will asked to participate in a targeted therapeutic trial with two differing treatment modalities offered. Both treatment strategies are comprised of well recognised elements which should be acceptable to all patients. All operative dental treatments offered would be carried out by a qualified dental surgeon working with a dental nurse. However, patients will be free to abstain from participation if they wish.

**Alternative procedures, if any, available to subjects: ?**

**Subjects:**

**a. What is the total number of subjects to be studied?** The power calculations have been made on summary OHIP-14 score data available from the UK Adult Dental Health Survey and on summary MNA data collected in studies performed in Cork. In both cases the null hypothesis is that patients receiving conventional treatment (Group 1) have a better outcome than those managed using functionally oriented treatment planning (Group 2). The alternative hypotheses are that Group 1 is no better than Group 2. Power was set at 80% with a one-sided 5% level of significance.

For OHIP-14, the standard deviation was 7.4 and the maximum difference allowed between groups is 4. For MNA, the standard deviation was 1.48 and the maximum difference allowed between groups is 0.80.

Based on these specifications, we need to recruit 44 patients to both treatment groups. To allow for a drop-out rate of up to approximately 30% (which may be pessimistic) during the study, we aim to recruit 65 patients per treatment group.

**b. How will subjects be chosen? (inclusion and exclusion criteria)**

Our target population will be Irish adults over the age of 65 years living in Cork and Kerry. We will recruit participants from two centres:

1. Patients attending the Geriatric Day Hospital facility at St. Finbarr’s Hospital, Cork. These are mostly over the age of 75 years, and may be considered “at risk” of developing nutritional problems associated with tooth-loss.

2. Patients attending for dental assessment and/or treatment at Cork University Dental School & Hospital. These patients would not pose the same level of risk for nutritional problems as those attending the Geriatric Day Hospital.

Our rationale for using two centres is 1) to broaden the age range of participants, and, 2) to increase the likelihood that the results of the study will be generalisable.

Patients will be excluded from the study if they were unable to speak English, illiterate or for any other reason which would impede their understanding of the questionnaires involved. Patients would also be excluded if they are physically unable to cooperate with the screening processes.

**Will there be payment to subjects? Yes No ✓**

**If so, how much?**

**Methods used to ensure confidentiality of data:**

Recorded data will not include patient’s names or addresses instead participants will be assigned a random numerical code to identify them. The only persons privy to the identities of the participants would be the investigators listed. Any clinical photographs taken as part of the study would not include identifiable characteristics of the participants.
